# High lithium concentration at delivery is a potential risk factor for adverse outcomes in breastfed infants: a retrospective cohort study

**DOI:** 10.1186/s40345-023-00317-4

**Published:** 2023-11-30

**Authors:** Essi Whaites Heinonen, Katarina Tötterman, Karin Bäck, Ihsan Sarman, Lisa Forsberg, Jenny Svedenkrans

**Affiliations:** 1 Department of Clinical Science, Intervention and Technology (CLINTEC), Div of Pediatrics, Karolinska Institutet, Blickagången 6A, 14157 Huddinge, Stockholm Sweden; 2 Department of Neonatology, Karolinska University Hospital, Stockholm, Sweden; 3 Sachs’ Children’s and Adolescents’ Hospital, Stockholm, Sweden; 4 Department of Neonatology, St Goran Hospital, Stockholm, Sweden; 5 Department of Clinical Science and Education Stockholm South Hospital, Karolinska Institutet, Stockholm, Sweden

**Keywords:** Bipolar disorder, Breastfeeding, Drug concentrations, Lactation, Lithium, Neonatal effects, Pregnancy, Psychiatry

## Abstract

**Background:**

Neonatal effects of late intrauterine and early postpartum exposure to lithium through mother’s own milk are scarcely studied. It is unclear whether described symptoms in breastfed neonates are caused by placental lithium transfer or postnatal exposure to lithium through breastfeeding. We aimed to investigate lithium clearance and neonatal morbidity in breastfed infants with high versus low serum lithium concentrations at birth.

**Methods:**

This retrospective study focused on breastfed infants to women treated with lithium during and after pregnancy, born between 2006 and 2021 in Stockholm, Sweden. Information on serum lithium concentrations and adverse neonatal outcomes was obtained from medical records. Neonatal symptoms and lithium clearance were compared between a high exposure group (HEG, lithium concentrations ≥ 0.6 meq/l) and a low exposure group (LEG, < 0.6 meq/l).

**Results:**

A total of 25 infant-mother dyads were included. Median lithium serum concentration at birth was 0.90 meq/l in the HEG as compared with 0.40 meq/l in the LEG (p < 0.05). The difference was still significant at follow-up (0.20 meq/l vs 0.06 meq/l, p < 0.05), despite reduction in maternal dose. The rate of neonatal symptoms was 85.7% in HEG and 41.2% in LEG (p = 0.08) at birth and 28.6% vs 11.8% at follow-up (p = 0.55). Furthermore, 28.6% of infants in HEG were admitted to neonatal care, vs 5.9% in LEG (p = 0.19). Two infants in the HEG had therapeutic lithium levels at follow-up. All infants with symptoms at follow-up were either in the HEG or exposed to﻿ additional psychotropic medication.

**Conclusions:**

Neonatal symptoms are common after late intrauterine lithium exposure, however transient, treatable and mostly mild. In this study, a high lithium concentration at birth was a risk factor for an increased lithium level at follow-up. Polypharmacy may constitute an additional risk factor. This study suggests that the late intrauterine exposure to lithium might add to the adverse effects in lithium-exposed, breastfed infants. Consequently we recommend breastfed infants with therapeutic lithium concentrations at birth to be followed up promptly to avoid lithium toxicity.

## Background

Bipolar disorder affects women of childbearing age. Lithium is commonly used to prevent manic and depressive episodes in bipolar disorder. The risk of relapse is substantial during, and especially directly after delivery (Viguera 2000). In addition to the increased risk of mortality and morbidity for the mother, the psychiatric illness can have a negative impact on the infant wellbeing and the mother and child bond, which can have far-reaching consequences (Walter et al. 2021; Oyetunji and Chandra 2020; Frayne et al. 2017). Therefore, discontinuation of treatment during pregnancy may imply significant risks both to the mother and the infant. It may be particularly important to ensure that patients with high risk of relapse, for example indicated by previous severe episodes, have adequate levels of lithium near delivery (Uguz et al. 2023).

On the other hand, intrauterine lithium exposure has been associated with several risks for the fetus. Significant placental passage of lithium has been documented (Uguz et al. 2023; Newport et al. 2005; Imaz et al. 2021). Cardiac malformations, such as Ebstein ‘s anomaly, have been associated to lithium exposure, even though the risk seems lower than previously suspected (Imaz et al. 2021; Patorno et al. 2017).

A wide range of neonatal complications are suggested to be connected to intrauterine lithium exposure. These include increased risks for prematurity, birth of a large for gestational age infant and lower APGAR scores, as well as higher rates of central nervous system and respiratory complications, jaundice, hypoglycemia, feeding challenges, neonatal diabetes insipidus, admission to neonatal intensive care unit, and thyroid and renal disorders (Newport et al. 2005; Boden et al. 2012; Hastie et al. 2021; Torfs et al. 2022; Poels et al. 2018; Forsberg et al. 2018; Schonewille et al. 2023; Frayne et al. 2018; Sagué-Vilavella et al. 2022). A relationship between neonatal symptoms and infant serum lithium concentration is suggested but not confirmed, and similar effects are also seen in infants exposed to maternal bipolar disorder without lithium medication (Newport et al. 2005; Boden et al. 2012; Schonewille et al. 2023; Sagué-Vilavella et al. 2022; Molenaar et al. 2021). No long-term neurodevelopmental effects have been reported in infants exposed to lithium in utero (Forsberg et al. 2018; Lugt et al. 2021; Poels et al. 2018).

Breastfeeding is strongly encouraged by WHO due to the health benefits for both the infant and the mother (World Health Organization 2009). Women who medicate with lithium have previously often been recommended to avoid breastfeeding (Poels et al. 2018; Galbally et al. 2018). Lithium transfers into breastmilk and previous clinical and animal studies have apart from high lithium concentrations in breastmilk and in the infant shown effects on infant central nervous system (CNS), thyroid and kidney function (Imaz et al. 2021; Heinonen et al. 2022; Viguera et al. 2007; Imaz et al. 2021; Imaz et al. 2021; Bogen et al. 2012; Newmark et al. 2019; Moretti et al. 2003; Ahmed et al. 2023). However, clinical cohorts with a total of around 100 breastfed infants have found breastfeeding safe during treatment with lithium under certain conditions (Imaz et al. 2021; Heinonen et al. 2022; Viguera et al. 2007; Imaz et al. 2021; Imaz et al. 2021). It is still unclear whether the early effects seen in lithium-exposed breastfed infants are caused by the lithium in mother’s own milk, or the late intrauterine exposure (Heinonen et al. 2022). Our previous study on the same cohort, presenting serum lithium concentrations and clinical effects during breastfeeding raised the question on clarifying the neonatal morbidities after intrauterine lithium exposure, and whether the outcomes that were found during follow-up could have been anticipated in the neonatal period (Heinonen et al. 2022). In this study, we aimed to describe the neonatal effects connected to late intrauterine lithium exposure and early postnatal exposure via breastfeeding. Furthermore, we aimed to relate any symptoms to the infants’ lithium serum concentrations.

## Methods

### Study design

This was a retrospective observational study of mothers treated with lithium and their newborn infants. Methods were reported according to the STROBE checklist (https://www.equator-network.org/reporting-guidelines/strobe/) for reporting observational cohort studies.

### Subjects

Study subjects were identified through diagnostic codes in medical records. All infants were born at either Karolinska University Hospital or Sachs´ Children’s and Adolescents’ Hospital, Stockholm, Sweden. Included infants were born from January 2018 to June 2021 at Karolinska and, from January 2006 to June 2021 at Sachs’ Children’s hospital. The parents were given a complete description of the study and asked for consent to participate by mail and telephone. Written informed consent from both guardians of the child was acquired for all participants.

### Clinical routine

The infant-mother pairs were observed and followed as per clinical routine at the time of birth. According to the routine established at Karolinska University Hospital in 2018, the maternal lithium dose was titrated by the psychiatrist during pregnancy and postpartum. At the time of delivery, the infant serum lithium concentration was measured in the umbilical cord. In conjunction with the neonatal screening at 48 h of age, tests for thyroid and kidney function were analyzed as well as a second serum lithium concentration. The results were assessed by a pediatrician before the infant was discharged. Infants were examined by a pediatrician at least once during the hospital stay and the results from the exam were registered systematically in the medical records. Infants with clinical symptoms or discrepant biochemical results had additional examinations and were admitted to neonatal care if necessary. Generally, women with severe psychiatric illness such as bipolar disorder, had a planned prolonged stay at the postnatal ward of 4–5 days. The routine also included a recommendation to stop the lithium intake at the start of contractions to decrease the risk of lithium toxicity for both mother and child and to continue with the pre-pregnant dose the day after delivery, but the adherence to routine varied. Maternal serum lithium concentrations were measured as trough values regularly during and after pregnancy. The dose was titrated by the mother’s psychiatrist.

The first follow-up visit of the infant was performed at 2–4 weeks of age and included infant serum lithium concentrations, tests for thyroid and kidney function and a clinical examination. At Sachs’ Children’s and Adolescents’ Hospital, the clinical routine and follow-up was similar, except for a variation in timing of the follow-up visits.

### Data collection

Main outcomes were presence of clinical symptoms at birth or at follow-up, and clearance of lithium in infant serum from birth to the follow-up visit. Secondary outcomes included infant-mother ratios of serum lithium concentrations, need for resuscitation within one hour from birth, admission to neonatal intensive care unit (NICU), prematurity, other neonatal morbidities, and recommendation to reduce breastfeeding at follow-up.

Infants with the first serum lithium concentration ≥ 0.6 meq/l were categorized into the high exposure group (HEG) whereas infants with concentrations < 0.6 meq/l were categorized into the low exposure group (LEG). In the three infants with missing lithium concentration measured in the umbilical cord, the concentration measured in serum at around two days of age was used as the grouping variable. The categorization was based on the lower limit for therapeutic concentration of lithium and previously published data (Newport et al. 2005; Nolen and Weisler 2013; Severus et al. 2008). All outcomes were compared between infants in the HEG and the LEG.

All data were collected in routine care as described above and retrieved from medical records for the study. Information on maternal health and illness during pregnancy and at delivery, smoking habits, use of alcohol, social factors and pharmacotherapy during pregnancy and breastfeeding were collected from the mothers’ healthcare records. Data on need for resuscitation or neonatal care and breastfeeding, the clinical routine examination of the newborn and the policlinical follow-up as well as infant concentrations of thyroid stimulating hormone, thyroid hormone, sodium, potassium and creatinine were collected from the infants’ healthcare records.

Serum lithium concentrations were analyzed by a colorimetric method, Modular P (2006 to 2016) or the Cobas 8000 c502 (2016 and later, both by Roche, Basel, Switzerland). The Karolinska University Laboratory has made a comparison of the instruments and demonstrated a good level of concordance. The estimated uncertainty of measurement was 10% for concentrations around 0.5 meq/L and 5% for concentrations around 1.4 meq/L. Maternal serum lithium concentrations were routinely measured as trough values during the study period, however, no individual information of the last maternal dose was available. Blood samples for infant serum lithium concentration were taken without correlation to the last maternal dose or last breastfeeding.

### Data management

The first infant concentration was used as a grouping variable for infant exposure level. Infant outcomes were compared between the exposure groups.

Placental transfer of lithium was analyzed by dividing serum lithium concentration in umbilical cord with the maternal concentration. The infant-mother ratio at follow-up was calculated by dividing the infant serum lithium concentration at follow-up with the maternal serum lithium concentration that was closest in time. A time difference of up to 14 days between the infant and maternal measurements was accepted, if the maternal dose was unchanged and the mother was considered to have stable serum lithium levels at the point of follow-up. The detection limit of lithium in serum was 0.05 meq/L. For measurements with undetectable lithium concentrations at follow-up, the value 0.04 was used in calculations, to avoid underestimation of the drug exposure.

Level of breastfeeding at discharge from maternity ward and at follow-up was categorized into exclusive breastfeeding or formula feeding in addition to breastfeeding. Infant symptoms at birth were defined as the binary variables preterm delivery, resuscitation within the first hour, admission to NICU, CNS-symptoms, jaundice, respiratory symptoms, renal symptoms (defined as increased serum creatinine levels or pathological levels of sodium or potassium in plasma) and thyroid symptoms (defined as altered levels of thyroid stimulating hormone and/or thyroid hormone). Length of stay at NICU and at maternity ward was analyzed as continuous variables.

Symptoms at the follow-up visit were categorized into the variables alertness, consolability, tone, sleep, feeding problems and poor infant growth. Growth was considered as poor if the infant had not regained their birth weight at visits before two weeks of age. For visits after two weeks, growth was considered as poor, if the infant had not gained an average of 15 g a day, equaling a loss of approximately half a standard deviation on the Swedish growth chart (Wikland et al. 2002). One infant with cardiac malformations was excluded from analyses of clinical symptoms.

All but four women used lithium sulfate. A 42 mg tablet of lithium sulfate (Lithionit®) contains 6 mg of the active substance of lithium (Lithionit Fass 2022). Four women were prescribed lithium carbonate, of which a 300 mg tablet contains 8 mg of the active substance of lithium (Carbonate 2022). Doses of the women with lithium carbonate were converted to doses of lithium sulfate accordingly (Sydvast and Litiumkompendiet. 2019).

### Statistical analysis

Descriptive statistics of continuous variables were presented as means, standard deviations (SD), medians and range. Categorical variables were presented as numbers of cases and percentages. Binary variables for any neonatal symptoms and any symptoms at follow-up were created. For difference between means of continuous variables in different exposure groups the Mann–Whitney U-test and the Related-Samples Wilcoxon Signed Rank Test were used. For categorical variables, hypothesis testing to assess differences between groups was performed by using Chi square and Fisher’s exact test. Pearson’s correlation was used to analyze the correlation between maternal and infant lithium concentrations at delivery, and the Spearman’s correlation test to analyze the correlation between maternal dose and infant concentrations. P-values < 0.05 were considered statistically significant. The statistical analyses were conducted by using SPSS version 28 (IBM Corporation, Armonk, New York, USA).

## Results

**Fig. 1:**
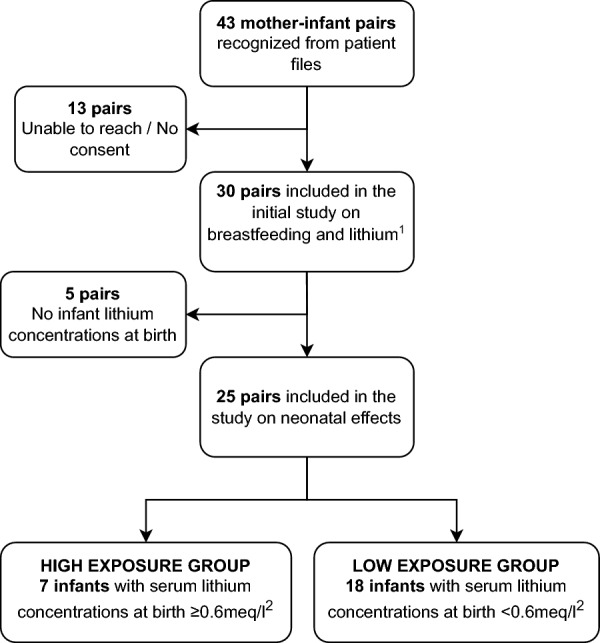
A flow chart of the study participants and exposure groups. ^1^ Heinonen et al. 2022. Lithium use during breastfeeding was safe in healthy full-term infants under strict monitoring (Heinonen et al. 2022). ^2^ Infant serum lithium concentration was measured in cord blood in all but three infants, for whom the lithium concentration measured at two days of age was used instead

A total of 43 infant-mother dyads were identified through diagnostic codes on the basis of the infant being exposed to lithium through mother’s own milk. Of these, 30 consented to be included in the follow-up study on effects of breastfeeding during lithium therapy (Heinonen et al. 2022). All but three infants were also exposed to lithium in utero, and for 25 of them, the serum lithium concentration measured in the umbilical cord or at the neonatal screening was available. These 25 infants were included in the analyses on the effects of intrauterine and breast milk exposure during the first weeks of life (Fig. 1).

### Infant characteristics

The 25 infants had a median (range) GA of 39 + 2 (35 + 2–41 + 0) weeks. The average (SD) birth weight was 3557 (407) g. All infants had APGAR at 5 min of 7 points or more. Seven infants were categorized into the high exposure group (HEG) and 18 to the low exposure group (LEG). One infant in the HEG was born preterm in week 35 + 2, all other infants were term. The same infant was large for gestational age, while all the other infants were appropriate for gestational age. One of the infants in the low exposure group was born with an atrioventricular septal defect. No other malformations were reported. A higher percentage of the infants were exclusively breastfed at follow-up (58.3%) compared with the time of discharge from hospital (45.8%). There was no difference in the level of exclusive breastfeeding between exposure groups. All other infants were partially breastfed. Initially, the mother’s need for sleep was the most common reason for giving additional formula. One infant was given formula due to prematurity and significant weight loss and one after an apparent life-threatening episode (ALTE) and therapeutic lithium concentrations at two days of age.

### Maternal characteristics

All but three mothers were diagnosed with bipolar disorder (Table 1). In 16 women, lithium treatment was initiated before pregnancy, and in seven during pregnancy (data not available for two mothers). Data on psychiatric symptoms at the time of delivery was available for 22 women. Of these, one woman in HEG and two in LEG were experiencing psychiatric symptoms at the time of delivery. The rest were reported to have no or mild symptoms.

**Table 1:** Background characteristics of the included mother-infant dyads

	High exposure group^a^		Low exposure group^b^		All infants	
	N	Mean (SD)	N	Mean (SD)	N	Mean (SD)
**Maternal characteristics**						
Maternal age	7	33.1 (6.5)	18	32.9 (5.7)	25	33.0 (5.8)
Pre-pregnancy BMI (kg/m2)	7	25.1 (4.7)	16	24.5 (3.6)	23	24.7 (3.9)

	N	n (%)	N	n (%)	N	n(%)
**Maternal diagnosis**						
Bipolar disorder 1	7	1 (14.3)	18	11 (61.1)	25	12 (48.0)
Bipolar disorder 2	7	3 (42.9)	18	5 (27.8)	25	8 (32.0)
Bipolar disorder unspecified	7	2 (28.6)	18	0 (0.0)	25	2 (8.0)
Other psychiatric diagnoses	7	1 (14.3)	18	2 (11.1)	25	3 (12.0)
**Other psychotropic medications**	7	5 (71.4)	16	11 (68.8)	23	16 (69.6)
Antidepressants	7	3 (42.9)	16	2 (12.5)	23	5 (21.7)
Antipsychotics	7	1 (14.3)	16	3 (18.8)	23	4 (17.4)
Anxiolytics and sedatives^d^	7	3 (42.9)	16	7 (43.8)	23	10 (43.5)
Psychostimulants	7	1 (14.3)	16	3 (18.8)	23	4 (17.4)
**Living with the father of the child**	7	7 (100.0)	16	13 (81.3)	23	20 (87.0)
**Employment**						
Full-time	7	1 (14.3)	16	6 (37.5)	23	7 (30.4)
Part-time	7	2 (28.6)	16	6 (37.5)	23	8 (34.8)
Not working or unemployed	7	4 (57.1)	16	4 (25.0)	23	8 (34.8)
**Tobacco use 3 months pre pregnancy**						
No smoking	6	3 (50.0)	16	11 (68.8)	22	17 (77.3)
1–9 cigarettes/day	6	1 (16.7)	16	2 (12.5)	22	3 (13.6)
> 10 cigarettes/day	6	1 (16.7)	16	3 (18.8)	22	4 (18.2)
Snuff	6	1 (16.7)	16	0 (0.0)	22	1 (4.5)
**Alcohol use 3 months pre pregnancy**						
Never	6	3 (50.0)	15	6 (40.0)	21	9 (42.9)
≤ 1 time/week	6	3 (50.0)	15	6 (40.0)	21	9 (42.9)
> 1 time/week	6	0 (0.0)	15	3 (20.0)	21	3 (14.3)
**Alcohol use in first trimester**						
Never	7	7 (100.0)	16	16 (100.0)	23	23 (100.0)
**Pregnancy and infant characteristics**						
** Delivery mode**						
Vaginal delivery	6	3 (50.0)	18	15 (83.3)	24	18 (75.0)
Instrumental delivery	6	1 (16.7)	18	3 (16.7)	24	4 (16.7)
Caesarean section, elective	6	1 (16.7)	18	0 (0.0)	24	1 (4.2)
Caesarean section, emergency	6	1 (16.7)	18	0 (0.0)	24	1 (4.2)
** Pregnancy complications**^e^	7	2 (28.7)	18	2 (11.2)	25	4 (16.0)
**Infant sex**						
Female	7	3 (42.9)	18	15 (83.3)	25	18 (72.0)

^a^Infant serum lithium concentration ≥ 0.6meqv/l

^b^Infant serum lithium concentration < 0.6meqv/l

^c^Independent sample t-test and Fisher’s exact tests were used to test group differences between the high exposure and the low exposure groups in continuous and nominal variables, respectively

^d^Zolpidem, Zopiclone, Oxycodone, Oxazepam, Propiomazine, Promethazine, Nitrazepam

^e^Pregnancy complications: hypertonia, polyhydramnios, elevated liver enzymes, hypothyroidism, hyperemesis gravidarum

The average (SD) dose of lithium sulfate or equivalent was 207 (97) mg/day at delivery and 197 (78) mg/day at follow-up, equaling 1109 (520) and 1055 (418) mg/day in lithium carbonate. The mean lithium dose at delivery was significantly higher in mothers with infants in the HEG than in the LEG (274 (98) vs 180 (85) mg of lithium sulfate a day, equaling 1468 (525) vs 964 (455) mg of lithium carbonate a day, p < 0.05). The difference in maternal lithium dose decreased until follow-up and was no longer statistically significant (222 vs 187 mg/day of lithium sulfate, equaling 1189 vs 1002 mg/day of lithium carbonate, p = 0.27, Table 2). Of the included women, 16 (70%) were also treated with other psychotropic drugs, whereof six with two or more drugs other than lithium. The level of polypharmacy was similar in both exposure groups(Table 1).

**Table 2:** Comparison of lithium serum concentrations and clinical characteristics between exposure groups

	High exposure group^a^				Low exposure group^a^				p^b^
	N	Mean (SD)	Median	Range	N	Mean (SD)	Median	Range	
Age at follow-up (days)	7	22.1 (11.2)	26	4–40	18	25.3 (13.6)	20	8–51	0.62
**Maternal dose of lithium sulfate (mg/d)**									
At delivery	7	274.0 (98.3)	252	168–448	18	180.3 (84.5)	168	21–336	< 0.05
At follow-up	7	222.0 (56.8)	210	140–294	18	187.1 (84.4)	168	21–336	0.27
**Lithium concentrations at birth**									
Infant umbilical cord (meq/l)	7	0.83 (0.24)	0.90	0.60–1.20	15	0.35 (0.14)	0.40	0.1–0.5	< 0.05
Infant serum at 2 days of age^c^	4	0.68 (0.25)	0.65	0.40–1.00	9	0.38 (0.12)	0.40	0.2–0.6	< 0.05
Mother (meq/l)	7	0.81 (0.34)	0.80	0.40–1.50	16	0.36 (0.20)	0.40	0.1–0.7	< 0.05
Infant/mother ratio^d^	7	1.10 (0.30)	1.10	0.70–1.50	13	0.95 (0.17)	1.00	0.70–1.30	0.34
**Lithium concentrations at follow-up**									
Infant (meq/l)	7	0.39 (0.41)	0.20	0.10–1.20	18	0.08 (0.07)	0.06	< 0.05 – 0.3	< 0.05
Mother (meq/l)	4	0.65 (0.06)	0.65	0.6–0.7	9	0.56 (0.21)	0.6	0.1–0.8	0.50
Infant/mother ratio	4	0.48 (0.46)	0.29	0.17–1.17	9	0.17 (0.14)	0.10	0.07–0.5	0.08

	N	n (%)			N	n (%)			p
**Exclusive breastfeeding**									
During hospital stay	7	3 (42.9)			18	8 (44.4)			1.0
At follow-up	7	4 (57.1)			17	10 (58.8)			1.0
**Clinical outcomes**^e^									
Symptoms at birth^f^	7	6 (85.7)			17	7 (41.2)			0.08
Symptoms at follow-up^g^	7	2 (28.6)			17	2 (11.8)			0.55
Recommended to reduce breastfeeding at follow-up	7	2 (28.6)			17	1 (5.9)			0.18

^a^Infants were assigned to study groups on the basis of the lower limit for therapeutic concentration, low exposure defined as infant lithium concentration < 0.6meqv/l and high exposure ≥ 0.6meqv/l

^b^Mann–Whitney U-test and Fisher’s exact tests were used to test group differences in continuous and nominal variables, respectively

^c^Measured in infant serum together with the neonatal screening test at median (range) 48.5 (36–62) hours of age

^d^Ratio between the lithium concentrations measured in the umbilical cord and in maternal serum at delivery

^e^The infant with cardiovascular malformation was excluded from the analysis of symptoms

^f^Specified in Table 3

^g^Symptoms at follow-up included poor infant growth in all 4 infants and in 2 of them, one in each exposure group, also tiredness

Three months before pregnancy, two (33.4%) women in HEG and five (31.3%) in LEG smoked on a daily basis. Three women (50%) in HEG and nine (60%) in LEG used alcohol. All women had discontinued use of alcohol and all but one in the LEG had discontinued smoking at admission to maternity care in the first trimester (Table 1). No illicit drug use was reported three months prior to or during pregnancy.

### Lithium concentrations

**Fig. 2:**
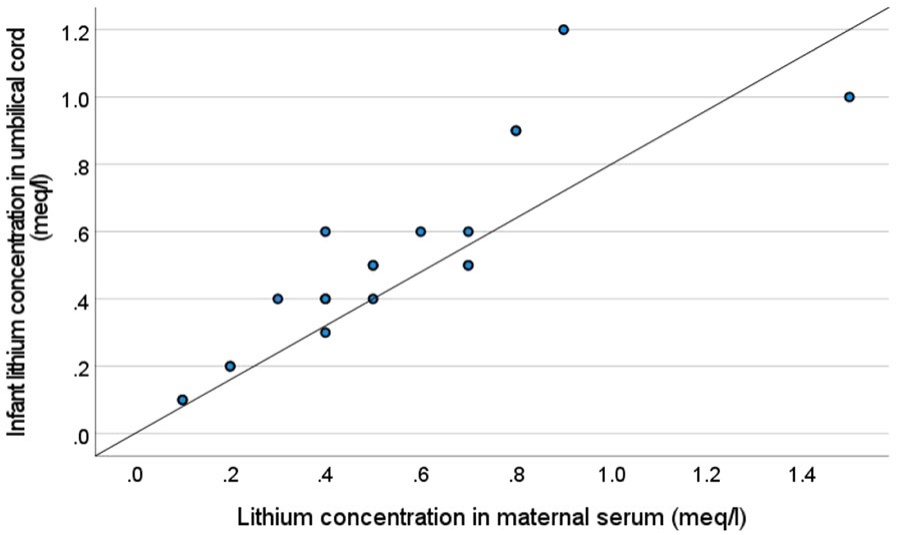
Scatterplot between infant and maternal lithium serum concentrations measured at delivery. There was a strong correlation between maternal and infant serum lithium concentrations at birth, R = 0.91, p < 0.05

Maternal serum lithium concentration at delivery was available for 23 mothers. The median maternal serum lithium concentration at delivery was 0.40 meq/l. The median infant/mother ratio at delivery was 1.0 with no difference between the groups (Table 2). There was a strong correlation between maternal and infant lithium concentrations measured in the umbilical cord, Pearson’s R 0.91, p < 0.05 (Fig. 2). Infant/mother ratio at follow-up was significantly lower than at birth (mean difference 0.89, p < 0.05).

**Fig. 3:**
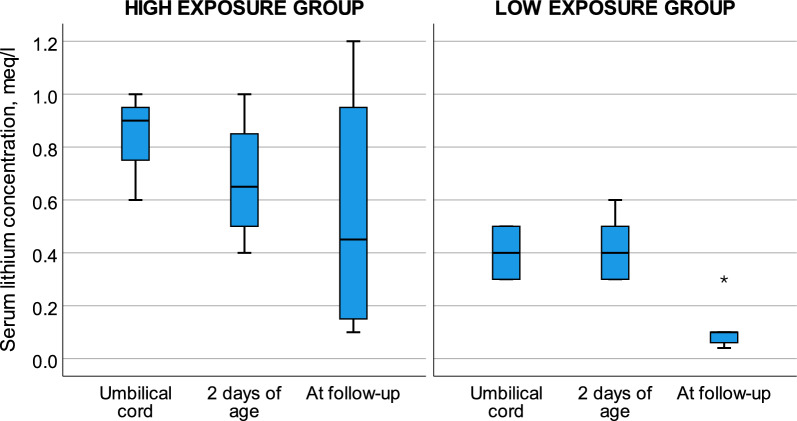
Boxplots of infant serum lithium concentrations. Infant serum lithium concentrations measured at birth were <0.6 meq/l in low exposure group (LEG) and  ≥ 0.6 meq/l in high exposure group (HEG). The concentrations were measured in the umbilical cord (n = 15 LEG, 7 HEG), at around 2 days of age (n = 9 LEG, 4 HEG) and at the first policlinical follow-up visit at median 20 and 26 days of age in LEG vs HEG (n = 18 LEG, 7 HEG)

Median infant serum lithium concentration measured in the umbilical cord was 0.45 meq/l, 0.9 meq/l in the HEG and 0.4 meq/l in the LEG, p < 0.05 (Table 2). Ten infants had both the serum lithium concentration analyzed in cord blood as well as a repeat measurement in infant serum at a median age of 48 h. There was no significant difference in the median serum lithium concentration between birth and at two days of age, 0.45 vs 0.40 meq/l, p = 0.20 (Fig. 3).

The median infant serum lithium concentration at follow-up was 0.10 meq/l. The median lithium concentration was significantly lower at follow-up than at birth, but higher in the HEG than in the LEG (0.20 vs 0.06 meq/l, p < 0.05, Fig. 3). There was no difference in median lithium concentration between exclusively and partially breastfed infants at 48 h of age, 0.40 vs 0.55 meq/l, p = 0.17, or at follow-up, 0.10 vs 0.08 meq/l, p = 0.37. In the LEG, the median serum lithium concentration in the exclusively breastfed infants was 0.07 meq/l, and 0.04 meq/l in the partially breastfed infants at follow-up, p < 0.05.

There was a significant correlation between the maternal dose of lithium sulfate and infant serum lithium concentration at birth, R = 0.41 (p < 0.05), but not at follow-up, R = 0.32 (p = 0.12).

### Infant clinical outcomes

At birth, 15 of 24 infants (62.5%) had clinical symptoms, 85.7% of the HEG and 41.2% of the LEG (p = 0.08), presented in Table 3.

**Table 3:** Neonatal symptoms in infants in high vs low exposure groups

Neonatal outcomes^a^	High exposure group		Low exposure group		p
	N	Outcome, n (%)	N	Outcome, n (%)	
Preterm delivery	7	1 (14.3)	17	0 (0.0)	0.29
Resuscitation within the first hour	7	2 (28.6)	17	3 (17.6)	0.61
Admission to NICU	7	2 (28.6)	17	1 (5.9)	0.19
CNS	7	2 (28.6)	17	2 (11.8)	0.55
Jaundice	7	2 (28.6)	17	2 (11.8)	0.55
Respiratory	7	3 (42.9)	17	3 (17.6)	0.31
Renal	3	1 (33.3)	5	0 (0.0)	0.38
Thyroid	2	1 (50)	2	1 (50.0)	1.00
Any complication	7	6 (85.7)	17	7 (41.2)	0.08

NICU: Neonatal intensive care unit, CNS: central nervous system. The infant with cardiovascular malformation was excluded from the analysis of symptoms

^a^Neonatal outcomes were divided into CNS symptoms (jitteriness, agitation, lethargy), jaundice, respiratory symptoms (apnea, need for CPAP and/or ventilation), renal symptoms (increased plasma creatinine levels) and thyroid symptoms (increased levels of thyroxine)

The most registered symptoms at birth were respiratory symptoms (apnea, labored breathing, need for CPAP and/or ventilation), seen in 25% of the infants with no statistically significant difference between HEG and LEG (Table 3). A fifth of all infants needed neonatal resuscitation within one hour after birth, with continuous positive airway pressure (CPAP) and/or ventilation, 29% in HEG vs 18% in LEG, p = 0.61. The CNS complications seen at birth were jitteriness, agitation and lethargy, seen in 16.7% infants, without a statistically significant difference between HEG and LEG. Thyroid levels were measured in five infants at birth and nine at follow-up. Two infants had elevated levels of thyroid hormone at birth, both normalized without treatment. Plasma creatinine levels were measured in nine infants at birth and 23 at follow-up. One infant had an elevated plasma creatinine level at birth, which was normalized at follow-up. No cases of hypoglycemia were detected.

Four infants (16%) were admitted to neonatal care, two in each exposure group, including the infant with atrioventricular septal defect in the LEG, who was excluded from the analyses of clinical symptoms. The length of stay at neonatal care was 1 day for the infants in the LEG and 0 and 2 days for the infants in the HEG. The median (range) length of stay at maternity ward was 4.5 (3–6) days for LEG and 4 (3–6) days for HEG. There was no statistically significant difference in the separate neonatal morbidities between the exposure groups (Table 3). A detailed description of the infants with possibly severe symptoms and/or high lithium levels is presented in Table 4.

**Table 4:** Description of infants with possibly severe symptoms and/or high lithium levels

ID, (HEG/ LEG)	Lithium concentration (meq/l)					Breastfeeding		Infant symptoms		Other psychotropic drugs	Potential cause of pathology
	MS	UC	IS	FU	MS-FU	At birth	At follow-up	At birth	At follow-up		
A (HEG)	0.8	0.9	0.6	0.7	0.6	Part	Part	Mild prematurity, large for gestational age, need for CPAP for one day. Initial weight loss of 15%	Poor weight gain at 11 days of age, recommended to increase formula	SSRI, anxiolytics	Combination of exposure to lithium and other drugs, lower kidney function due to prematurity and dehydration due to large initial weight loss
B (HEG)	0.8	0.9	0.7	1.2	–	Part	Part	Found cyanotic in the bed at maternity ward at 2 days of age but recovered without respiratory support. Observed for 2 days at NICU	No clinical symptoms but advised to stop breastfeeding due to high lithium concentration at 29 days of age	Anxiolytics when needed	Combination of pre- and postnatal exposure together with some unknown inborn fragility in the infant causing both the cyanotic spell and the high lithium concentration at follow-up?
C (HEG)	1.5	1.0	1.0	0.2	0.7	Excl	Excl	Jittery at birth, blood glucose checked, normal. Elevated plasma creatinine level of 84 μmol/l at 48 h of age	Normalized plasma creatinine, 48 μmol/l, but jaundiced, tired and poor weight gain at 9 days of age	SSRI, central stimulants, anxiolytics	Toxic maternal serum lithium concentration and potentially maternal dehydration causing increased infant creatinine level at birth. However, the effects at follow-up might rather have been caused by the polypharmacy
D (HEG)	0.9	1.2	–	0.1	–	Excl	Excl	No clinical symptoms at birth	No clinical symptoms at 26 days of age	Antipsychotics	High infant serum concentration at birth caused by high intrauterine exposure, without any clinical consequences and good clearance at follow-up
E (LEG)	0.5	0.5	0.4	< 0.05	0.6	Excl	Part	Jittery at birth, blood glucose checked × 3, normal	Tired with poor weight gain at 19 days of age, recommended to introduce formula	Anxiolytics	Jitteriness and poor weight gain may have been caused by the intrauterine lithium exposure as well as the polypharmacy
F (LEG)	0.4	0.3	0.4	0.1	0.5	Part	Part	Cyanotic with respiratory distress at 25 min of age. Admitted to NICU, treated with CPAP for 1 day. Elevated thyroid hormone level, 47 pmol/l	Poor weight gain at 12 days of age. Normalized thyroid levels	SSRI, antipsychotics	Both the need for respiratory support at birth and the poor weight gain may be effects of polypharmacy rather than lithium exposure only
G (LEG)	0.2	–	0.2	< 0.05	0.1	Part	Part	Need for CPAP briefly from one minute of age, no need for admission to NICU	No symptoms at the follow-up at eight days of age	Central stimulants	Need for respiratory support initially likely to be related to the instrumental delivery rather than exposure to low dose lithium
H (LEG)	0.2	0.2	–	0.1	–	Excl	Excl	Pale and hypotonic shortly after birth. Receives treatment with CPAP briefly, after that no need for admission to NICU	No symptoms at the follow-up at 29 days of age	–	Short need for respiratory support due to other factors than the low exposure to lithium

HEG: High exposure group, serum lithium measured at birth ≥ 0.6 meq/l, LEG: Low exposure group, serum lithium measured at birth < 0.6 meq/l. MS: Maternal serum at delivery, UC: Umbilical cord, IS: Infant Serum at 2 days of age, FU: Infant serum at follow-up, MS-FU: Maternal serum at follow-up CPAP: Continuous positive airway pressure, NICU: Neonatal intensive care unit, Excl: Exclusive, Part: Partially, SSRI: selective serotonin reuptake inhibitors, anxiolytics: benzodiazepines, antihistamines and other anxiolytic and sedative drugs

### Symptoms at follow-up

The first follow-up visit was at an average (SD) age of 24 (12.5) days, with no statistically significant difference in time to first visit between the exposure groups (Table 2). Four infants were categorized as having clinical symptoms at follow-up. These included poor infant growth (4 infants) and tiredness (2 infants, one from each group). In addition, two infants in the HEG and one in the LEG were recommended to reduce breastfeeding due to high serum lithium concentrations and/or clinical symptoms (Tables 2, 4). There was no difference in clinical symptoms between exclusively and partially breastfed infants at follow-up (14.3 vs 20.0%, p = 1.0). There was a significant reduction in symptoms to the time of follow-up in both groups, to 28.6% symptoms in the HEG, and 11.8% in the LEG, but no statistically significant difference in symptoms between HEG and LEG (p = 0.55).

### Symptoms in relation to polypharmacy

Of the 15 infants to mothers treated with other psychotropic drugs in addition to lithium, 8 (53.3%) had neonatal symptoms, compared to 4 (57.1%) of the 7 infants to mothers treated with lithium in monotherapy (p = 1.0). At follow-up, all 4 infants with poor growth had mothers treated with other psychotropic drugs as well, equaling 26.7% of the infants of the polypharmacy group, vs 0 infants in lithium only group, p = 0.26.

## Discussion

This study of 25 mother-infant dyads is the first aiming to analyze the neonatal effects of lithium exposure during late pregnancy as well as via mother’s own milk during the first weeks of life. We found that mild neonatal symptoms were common, however, the symptoms were transient, easily treated, and no adverse effects needed immediate intervention after discharge from the postnatal wards. No severe symptoms were described at follow-up, and a majority of the infants had a significant reduction in serum lithium concentrations during the first weeks of life, although they were fed mother’s own milk. However, some infants with high intrauterine exposure still had therapeutic levels of lithium at follow-up. These infants need to be recognized at birth and followed rigorously and according to a predefined protocol.

These results are particularly important since breastfeeding traditionally has been discouraged from for mothers treated with lithium (Poels et al. 2018; Galbally et al. 2018). Breastfeeding is documented to improve the psychological and physical health of both mother and infant, and is shown especially meaningful for the mental well-being of mothers with severe mental illness (Anderson 2018; Chowdhury et al. 2015; Baker et al. 2021; Tucker and O’Malley 2022). While this is true for some women, breastfeeding can also cause disturbed sleep, and an individual approach where pros and cons are carefully evaluated is necessary. Where the need for undisturbed sleep is judged important for preventing relapse, abstaining from breastfeeding during night-time may be the best option if there is a partner or another family member who can provide necessary support at night. A psychiatrically stable mother in remission is always the best for the infant, and hence, breastfeeding will never be suitable for all new mothers with severe mental illness (Galbally et al. 2018).

The results in this study suggest that the risk of adverse events increases with higher late intrauterine exposure. No statistical difference could be found in the overall level of symptoms. There was however a trend towards more neonatal symptoms in the high exposure group, in line with previous findings by Newport et al. (2005). Furthermore, at follow-up, none of the infants in the low exposure group had high lithium concentrations, and all unexpected serious events, like ALTE and toxic lithium levels, were found in the HEG (Tables 2, 4). The importance of late intrauterine exposure is further highlighted by the difference in infant serum lithium concentrations at follow-up between the low exposure group and high exposure group, indicating a slower clearance of lithium in the high exposure group. However, the higher lithium concentrations in the HEG at follow-up could also be explained by continuous high exposure via mother’s own milk, even though the lithium doses of the mothers in the HEG were reduced postpartum. A third possible explanation for the continuously higher infant serum lithium concentrations in the HEG is reduced renal function in newborn infants, as lithium is solely eliminated through renal excretion (Clark et al. 2022). Some infants exposed to high lithium levels at birth may not have the capacity to excrete the additional lithium from the mother’s own milk, resulting in therapeutic levels at follow-up We suggest that infant symptoms in the first month of life may be caused a combination of intrauterine exposure and through mother’s own milk. On the other hand, a study by Molenaar et al. 2021 suggested that there is no correlation between lithium concentration at birth and neonatal symptoms, implicating that more studies are needed to confirm the results. Some guidelines recommend that lithium is temporarily stopped at the start of labor (The Management of Bipolar Disorder Working Group 2010; Institutes and of Excellence (NICE). 2014; ). On the other hand, an adequate lithium level towards the end of the pregnancy is often emphasized as an important measure to prevent serious postpartum illness (Poels et al. 2018; Molenaar et al. 2021). Nevertheless, this finding highlights the importance of a structured follow-up of infants exposed to lithium through mother’s own milk, and high intrauterine exposure may constitute an additional risk.

For comparison between high and low exposure, we placed the cut-off at 0.6 meq/l. This cut-off was chosen to reflect the lower limit of therapeutic interval of lithium, as well as to repeat the comparison in a previous report (Newport et al. 2005). Another study suggested a limit of 0.3 meq/l as a safe infant lithium concentration during breastfeeding (Imaz et al. 2021). In our study, all infants exposed to lithium in monotherapy and with lithium below 0.3 meq/l were without symptoms at follow-up. However, two infants with low serum lithium concentrations but also several other psychotropic medications showed symptoms at follow-up (patient E and F, Table 4). In fact, all infants with symptoms at follow-up were exposed to additional psychotropic drugs. Therefore we conclude that polypharmacy may be an additional risk factor for adverse effects and suggest rigorous monitoring of infants exposed to polypharmacy.

In the antenatal consultations within our follow-up program, we try to emphasize the importance of the possibility to decrease lithium exposure when needed. For this, we recommend all parents to give at least one bottle feed a day to be able to reduce breastfeeding if necessary. In the LEG, the serum lithium concentration was significantly lower in the partially breastfed infants than in the exclusively breastfed infants, suggesting that introduction of formula is an efficient method to bring down the infant serum lithium concentration if needed.

The strengths of this study include detailed descriptions of neonatal morbidity and low numbers of missing data on infant lithium concentrations measured at both birth and follow-up. Another strength, as well as a limitation, is the observatory nature of the study, including infants exposed to extensive polypharmacy. This could overestimate the neonatal morbidity connected to lithium exposure but describes on the other hand the overall risks in these patients. The generalizability of the results is limited by the follow-up program only including psychologically stable and highly motivated women, and healthy infants. Consequently, the results should not be applied to all women with bipolar disorder treated with lithium. Furthermore, the need for undisturbed sleep may be a reason to recommend the mother to abstain from breastfeeding. The lack of a control group not exposed to lithium is a limitation, especially when interpreting the significance and cause of the clinical symptoms. Lastly, a larger cohort would have strengthened the implications and conclusions drawn from the results.

Our study confirms that lithium passes freely across the placenta with lithium concentrations equilibrated between maternal and fetal circulations. This and the descriptions of neonatal symptoms of intrauterine lithium exposure are concurrent with previous studies (Newport et al. 2005; Hastie et al. 2021; Schonewille et al. 2023). In our study, milder respiratory symptoms treated with CPAP according to routine were the most common, but one case of ALTE was also described. As short-term ventilatory support with CPAP is very common, and no more advanced resuscitation efforts were needed, these symptoms were assessed as transient and easily treated. This study supports dose-dependency of the neonatal symptoms seen in the study from Newport et al. but as there is also recent evidence stating the contrary, this question remains to be solved by larger cohorts (Newport et al. 2005; Molenaar et al. 2021).

A recent study suggested that exclusive breastfeeding could be safe during treatment with lithium in monotherapy, without accumulation of lithium in the infant (Imaz et al. 2021). Our data support this statement and suggest that breastfeeding is possible when the mother is psychiatrically stable and well informed, and when a structured follow-up is available, possibly even when exposed to polypharmacy. However, it is important that the decision whether to allow breastfeeding is individualized and that all risk factors, such as high infant lithium concentration, polypharmacy and degree and stability of the underlying maternal condition, are considered and that the follow-up is adjusted accordingly.

## Conclusion

Our study suggests, that infants with late intrauterine exposure to lithium have a high risk of neonatal symptoms, especially when exposed to higher levels of lithium. Although common, the symptoms are transient, treatable, and mostly mild. Furthermore we suggest, that high lithium levels at birth constitute a risk factor for continuous high lithium levels in breastfed infants at follow-up. Polypharmacy may also imply an additional risk factor for clinical symptoms. Although all infants exposed to lithium through breast milk warrant clinical follow up, we suggest that those with risk factors should be followed up even more promptly to avoid lithium toxicity. Future studies should focus on identifying the lithium exposed infants in need of a more rigorous follow-up and possibly differentiate this group from those who can be breastfed safely with only minor precautions.

## Data Availability

The data that support the findings of this study are not openly available due to reasons of sensitivity and are available from the corresponding author upon reasonable request. Data are located in controlled access data storage at Karolinska Institutet.

## Abbreviations

ALTE: Apparent Life-Threatening Episode
CNS: Central Nervous System
CPAP: Continuous Positive Airway Pressure
HEG: High Exposure Group
LEG: Low Exposure Group
NICU: Neonatal Intensive Care Unit
